# Excimer Laser System: The Revolutionary Way to Treat Psoriasis

**DOI:** 10.7759/cureus.50249

**Published:** 2023-12-09

**Authors:** Abdullah Alyoussef

**Affiliations:** 1 Internal Medicine, Faculty of Medicine, University of Tabuk, Tabuk, SAU

**Keywords:** side effects of excimer laser, psoriasis area and severity index (pasi), psoriasis, protocols of excimer laser, phototherapy, excimer laser

## Abstract

Psoriasis is a chronic, autoinflammatory skin disease that affects approximately 100 million people worldwide. It is a systemic disease characterized by scaly, red patches on the skin and can also affect the joints. Psoriasis can significantly affect a person's physical and mental health. The prevalence rate of psoriasis may vary depending on the specific population studied and the diagnostic criteria used. Phototherapy is a safe and effective treatment for psoriasis that involves exposing the affected skin to specific wavelengths of light. It can be used alone or with other treatments for severe psoriasis. However, clinicians must choose the right light source for each type of psoriasis and monitor the patient closely during treatment to avoid adverse events. The 308 nm excimer laser is a widely used device in dermatology for treating several skin conditions, including psoriasis. Although the excimer laser can treat various dermatologic diseases, this study will focus only on its effectiveness in treating psoriasis. This study will review the use of an excimer laser, its protocol, and its side effects.

## Introduction and background

Psoriasis is a chronic auto-inflammatory skin disease that affects both the skin and joints. It is a common condition, affecting around 100 million people worldwide [1]. The prevalence of this disease is on the rise. According to data collected from various countries in the USA and Europe, the estimated prevalence rate ranges between 0.91% and 8.5% [2]. It's important to note that the prevalence rate can vary depending on the specific population studied and the diagnostic criteria used.

Psoriasis is a skin condition that causes scaly, red patches on the skin. In severe cases, it can also affect the joints, making daily activities difficult, and affecting a person's physical and mental health significantly. Patients with psoriasis often suffer from other health problems like psoriatic arthritis, inflammatory bowel disease, metabolic syndrome, cardiovascular disease, psychological disorders, atherosclerosis, and chronic obstructive pulmonary disease [3]. This condition mostly affects young adults, with two-thirds of patients developing the disease before the age of 40. One-third of patients develop the condition in childhood, while 57% suffer moderate to severe psoriasis. In conclusion, psoriasis is not just a skin disease but a systemic one, which means that it can affect other organs and systems in the body. It is linked to various comorbidities, including a higher risk of early atherosclerosis in patients, the buildup of plaque in arteries leading to cardiovascular diseases. Additionally, psoriasis has been associated with other inflammatory conditions, such as inflammatory bowel disease, which indicates that it may have a broader impact on the body beyond just the skin and joints [4].

Psoriasis is a chronic autoinflammatory disease that can present itself in various clinical forms. The most common condition is plaque psoriasis, which affects most patients. It is characterized by raised, red, and scaly patches on the skin. Another type of psoriasis is guttate psoriasis, typically diagnosed in children after an acute streptococcal episode. It appears as small, drop-like lesions on the skin. Amicrobial bumps characterize the pustular form of psoriasis and can affect various body parts, locally or generally. The erythrodermic form of psoriasis is a severe and life-threatening type that results in widespread redness and inflammation of the skin [2]. Psoriatic lesions can appear on the scalp, palms, soles, genitals, and nails. Nail involvement is common in psoriatic arthritis patients [5].

Although the exact mechanisms behind psoriasis are not yet fully understood, it is known that the condition is characterized by the excessive production and accumulation of skin cells, known as keratinocytes. These cells undergo abnormal differentiation, leading to the formation of thick, scaly patches on the skin. Psoriasis is also associated with infiltrating various immunoinflammatory cells, including T cells, dendritic cells, and neutrophils, into the affected skin [6]. Psoriasis is believed to be caused by a positive feedback loop between activated immune cells and hyperproliferative epidermal keratinocytes, which perpetuates inflammation and worsens the condition. Interestingly, the pathological changes observed in psoriasis are similar to those seen in cancer, where the uncontrolled proliferation of cancer cells leads to tumor growth. This suggests that some common factors may be shared between psoriasis and cancer, such as dysregulated cell signaling pathways and immune dysfunction. Further research is needed to fully understand the complex interplay between these factors and their contribution to the development and progression of psoriasis [4].

**Figure 1:**
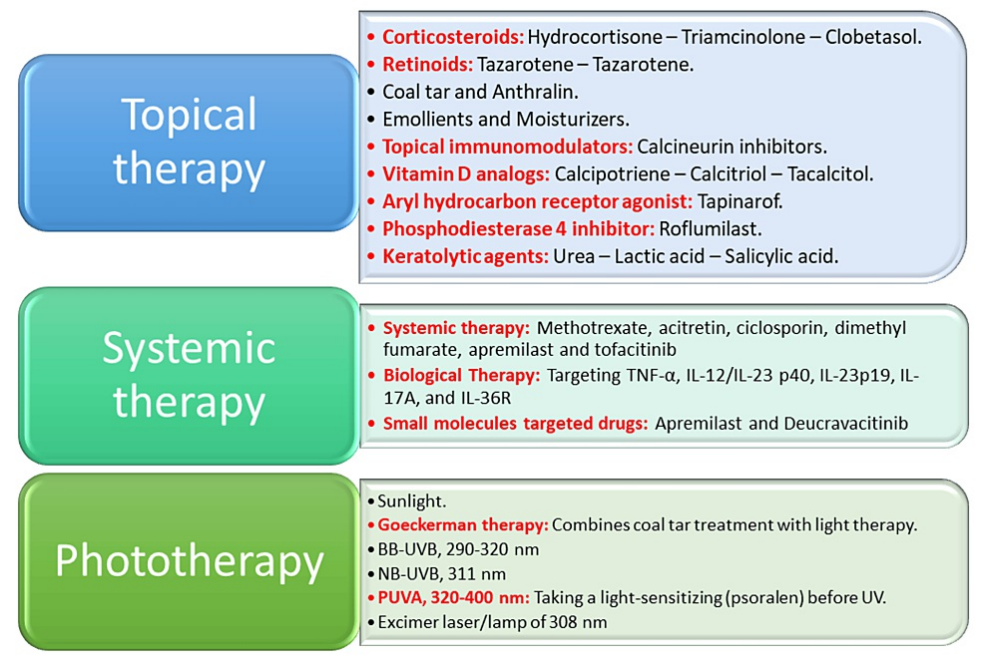
Schematic presentation of available therapies for psoriasis. BB-UVB, broadband ultraviolet B light; IL, interleukin; NB-UVB, narrowband ultraviolet B; PUVA, psoralen plus ultraviolet A; TNF-α, tumor necrosis factor-α. This image was created by the author of this study.

Psoriasis is an incurable chronic condition that requires lifelong treatment. The treatment of psoriasis depends on various factors, such as the severity of the disease, its location, and any accompanying conditions. Topical therapy, involving the use of corticosteroids, vitamin D analogs (such as calcipotriene, calcitriol and tacalcitol), retinoids (such as tazarotene and tazarotene), anthralin and topical immunomodulators as calcineurin inhibitors, is the preferred treatment for patients with mild psoriasis [7]. Psoriasis is a common skin condition that can cause dry and irritated skin. Emollients are an effective first-line treatment for psoriasis because they help to soothe and moisturize the skin. Keratolytic agents like salicylic acid can also be used to treat psoriasis by reducing the cohesion between skin cells and making them softer and easier to remove. In addition to these treatments, there are also new therapeutic agents available for psoriasis, such as tapinarof, which is an aryl hydrocarbon receptor agonist, and roflumilast, which is a phosphodiesterase 4 inhibitor [8]. Conventional oral systemic therapies, such as methotrexate, acitretin, ciclosporin, dimethyl fumarate, apremilast, and tofacitinib, are the treatment options for those with moderate to severe psoriasis [9]. Biological therapies that target tumor necrosis factor α (TNFα), interleukin (IL)-12/IL-23 p40, IL-23p19, IL-17A, and IL-36R are increasingly used for patients with moderate to severe, refractory, and special types of psoriasis [10]. These biologics are currently used in the clinical treatment of psoriasis and are proven to be more effective than non-biological treatments. Oral small-molecule targeted drugs offer more convenience to patients, lower healthcare costs, and improved quality of life compared to biologics. Currently, two oral small-molecule targeted drugs, apremilast, a phosphodiesterase 4 (PDE4) inhibitor, and deucravacitinib, a tyrosine-protein kinase 2 (TYK2) inhibitor, are available for treating psoriasis (Figure 1) [11].

Psoriasis is characterized by thickening of the epidermis due to the proliferation of keratinocytes. This happens because immune cells, particularly T lymphocytes, release several cytokines that cause an accelerated epidermal turnover. In psoriasis cases, dilated and elongated blood vessels are present in the papillary dermis, and there is a perivascular infiltrate consisting of lymphocytes and macrophages [2].

## Review

Psoriasis Area and Severity Index (PASI)

Psoriasis severity can be determined using a scoring system called the PASI score. This score evaluates the extent of skin involvement and symptom severity and ranges from 0 to 72. The score is calculated by assessing the body surface area affected by psoriasis and assigning a score based on the skin lesions' redness, thickness, and scaling. A score of 0 means no psoriasis, while 72 indicates the most severe form of the disease. When evaluating the PASI score, it is essential to note that the score is subjectively evaluated by the doctor's visual assessment of the skin lesions. Therefore, having the same doctor perform the evaluation consistently is crucial to ensure accurate PASI scoring over time. A PASI score up to 10 is considered mild psoriasis, while a score above 10 indicates moderate to severe psoriasis [12]. The PASI score is an essential tool for monitoring psoriasis treatment effectiveness and making informed treatment decisions. It helps doctors evaluate how well a patient is responding to treatment and whether any changes need to be made.

The use of phototherapy in psoriasis

Psoriasis is a skin condition that can be treated with phototherapy. This is a safe and effective procedure that doesn't have any systemic side effects. Phototherapy involves exposing the affected skin to specific wavelengths of light, which can slow down the growth of skin cells and reduce inflammation. Phototherapy can be used alone or in combination with biological agents to treat severe psoriasis. When selecting the proper light source for phototherapy, it's important to consider the patient's skin type, age, medical history, and the severity of psoriasis [13]. Clinicians need to choose the right type of laser or light and adjust the parameters to prevent unwanted side effects, such as skin burning, photoaging, and erythema. During treatment, clinicians should closely monitor the patient and make any necessary adjustments to the light source or parameters to ensure the treatment remains safe and effective.

During UV treatment, it is important to note that the dose-escalation is limited by the lower tolerance of normal skin to UV radiation as compared to psoriatic plaques. This means that the amount of UV radiation that can be safely applied to the skin without causing harm to the healthy skin is lesser than that which can be applied to the psoriatic plaques. As a result, the treatment needs to be carefully monitored and adjusted to ensure that the psoriatic plaques are effectively treated while minimizing any potential damage to the surrounding healthy skin [14].

Over the last few decades, phototherapy for treating psoriasis has seen significant progress. The first form of phototherapy to be developed was broadband ultraviolet B light (BB-UVB, 290-320 nm). However, it was later replaced by narrowband ultraviolet B (NB-UVB, 311 nm) as it was found to be more effective in treating psoriasis [15]. In 1997, the excimer laser/lamp of 308 nm was introduced as a monochromatic UVB source for psoriasis treatment. Compared to other treatments, the excimer has several advantages, such as targeting affected skin while sparing unaffected skin and providing high doses to the skin involved. Studies have also shown that the 308 nm excimer lamp is as effective in clearing psoriasis as the excimer laser [16].

There are different options available for phototherapy, one of which is Goeckerman therapy. In this approach, coal tar treatment is combined with light therapy to make the skin more responsive to ultraviolet B light. Although Goeckerman therapy is considered safe, using tar may cause side effects such as contact dermatitis and mild local burning due to tar hypersensitivity [17]. Psoralen plus ultraviolet A (PUVA) photochemotherapy involves using psoralens, a type of phototoxic plant-derived compound, along with exposure to ultraviolet A radiation. PUVA effectively treats several skin conditions, including psoriasis, mycosis fungoides, eczema, vitiligo, and graft-versus-host disease. However, the long-term side effects of PUVA are skin dryness, freckling, and wrinkling. There is also an increased risk of sun-related skin cancer later in life, particularly with the higher doses of PUVA [18].

Both NB-UVB and excimer laser are used as first-line therapy for stable plaque psoriasis. The decision to use a particular treatment is typically based on the individual patient's condition, medical history, and other factors. The effectiveness of each treatment may vary depending on the patient's response to therapy. Overall, these phototherapy options have significantly improved psoriasis management and provided patients with more treatment options for this chronic skin condition.

The 308 nm excimer laser

The 308 nm excimer laser is commonly used in dermatology to treat various conditions, including psoriasis, vitiligo, alopecia areata, atopic dermatitis, hypopigmented disorders, cutaneous T-cell lymphoma, other lymphoproliferative disorders, granuloma annulare, Langerhans cell histiocytosis, lichen planus, and localized scleroderma [19]. Its name, "excimer," comes from "excited dimer," which refers to the combination of noble gas xenon and halogen chloride gas (XeCl) used in this device. The device dissociates these excited dimers to produce a 308 nm ultraviolet (UV) monochromatic coherent wavelength within the UVB spectrum [20]. While the excimer laser can treat various dermatological conditions, this study will focus specifically on its effectiveness in treating psoriasis.

The 308 nm wavelength emitted by the excimer laser is effective in inducing apoptosis in keratinocytes and T lymphocytes, which are the two major cell types involved in the pathogenesis of psoriasis [19]. As soon as the skin absorbs the 308 nm wavelength, it leads to DNA damage in the keratinocytes and T lymphocytes, which activates the tumor suppressor gene p53 and decreases the proto-oncogene Bcl-2. The reduction of Bcl-2 in turn causes cell cycle arrest in both keratinocytes and T lymphocytes, thereby interrupting the psoriatic disease cycle initiated by the activated T lymphocytes. This process effectively disrupts the positive feedback loop that perpetuates psoriasis. Moreover, high doses of excimer laser treatment can also decrease the number of pathogenic memory/effector T cells that infiltrate the lesional epidermis and dermis in psoriatic lesions, thereby reducing inflammation and assisting in the clearance of psoriatic lesions [21].

Laser therapy can be used to treat moderate to severe psoriasis that affects less than 10% of the body surface. This treatment offers advantages over conventional phototherapy in hard-to-treat areas such as the nose, ears, and palpebral region. Laser therapy involves lower UV exposure, shorter treatment times, and the ability to focus on the affected skin while avoiding unaffected areas [20].

Safety of the excimer laser

When compared to the traditional narrow-band UV therapy, the treatment with the excimer laser has several advantages. Firstly, it requires fewer sessions, which results in less cumulative UVB exposure. This aspect is particularly significant as it reduces the risk of skin cancer and improves patient compliance [20]. Secondly, the excimer laser can target hard-to-reach areas and deliver site-specific dosing, which is impossible with traditional narrow-band UV therapy. Thirdly, energy levels can be adjusted according to the patient's needs during treatment. This personalized approach ensures better outcomes and reduces the risk of adverse reactions [22].

Protocols of the excimer laser

The 308 nm excimer laser has undergone significant advancements, leading to the development of several effective treatment protocols for psoriatic lesions that are now widely used in clinical practice. Among these protocols, three approaches have emerged as particularly effective in achieving significant improvements in the PASI score while requiring fewer treatments. These include the minimal erythema dose (MED) protocol, the induration protocol, and the minimal blistering dose (MBD) protocol. Each of these protocols involves a slightly different approach to applying the 308 nm excimer laser to achieve optimal results while minimizing the risk of adverse effects [23]. Staying updated with the latest advancements in laser technology and treatment protocols will enable dermatologists to provide their patients with the most effective and safe treatments available.

MED protocol

The initial step to determine the MED required for treating psoriasis involves testing healthy skin. During this process, the aim is to identify the amount of millijoules that results in a faint pink mark with minimal redness [24]. Typically, this involves two visits to calibrate the MED, followed by 12 treatment sessions until PASI-75 is achieved. It should be noted that precise information regarding treatment dosages in millijoules is not widely available. Most studies only report cumulative doses at the end of the treatment course. However, Asawanonda et al. conducted a study that extrapolated dosing in millijoules from a mean MED of 203.3 MJ, which correlated to a dose of 3.248 MJ. These details provide a better understanding of the process involved in determining the MED for psoriasis treatment [25].

Induration protocol

Taneja and their team have come up with a groundbreaking protocol that utilizes induration to determine treatment dosing for psoriasis patients. The protocol involves administering an initial dose based on the induration component of the modified PASI and subsequently adjusting doses based on changes in induration. Compared to the traditional MED protocol, the induration method allows clinicians to customize the dose as plaques respond to treatment, resulting in a more personalized and effective treatment plan. This method has shown remarkable success rates, with psoriasis treated with the induration-based protocol achieving PASI-75 after an average of only ten treatments [26]. This underscores the potential of this protocol to improve patient outcomes and offer a more personalized approach to psoriasis treatment. By incorporating this method into clinical practice, clinicians may achieve better long-term control of psoriasis symptoms and improve patient's quality of life.

MBD protocol

A report by Debbaneh et al. has introduced a protocol that employs MBD for excimer laser treatment. The protocol involves identifying the minimum dose required to induce blistering in the patient and then administering subsequent treatments at doses lower than MBD. The objective is to deliver the maximum possible dose to the affected area without causing blistering [27]. In one case study, doses ranging from 300 to 1700 mJ were tested on the patient, with MBD being determined at 1500 mJ. A starting dose below the blistering threshold was set at 1300 mJ. The patient underwent sub-blistering doses of 800 mJ for 12 days and showed significant improvement in their condition, achieving PASI-75 at the one-week follow-up [28].

Side effects of the excimer laser

Adverse reactions related to phototherapy are quite minimal and comparable to those observed with other types of phototherapy. These reactions consist of erythema, blistering, hyperpigmentation, erosions, and hypopigmentation [29]. However, the cumulative radiation doses associated with this therapy are less than those of UVB narrow-band treatment. Compared to UV phototherapy, 308 nm excimer laser therapy focuses on the affected skin, requiring a lower exposure period. When it comes to treatment safety, a previous clinical trial by Peng and colleagues in 2021, the group that received a high dose had more adverse reactions. However, it was discovered during follow-up that the patients quickly recovered by using an external skin repair agent after completing the therapy. The incidence of adverse reactions may be related to the thickness of the skin and the level of inflammation. Therefore, it is crucial to evaluate the skin and lesion type before selecting the dose. Interestingly, the high-dose treatment may cause opposite effects due to the higher risk of adverse reactions [30].

## Conclusions

Psoriasis is a skin condition that affects many people worldwide. Although there are several treatment options available, some patients may not respond well to traditional therapies. In such cases, the excimer laser has emerged as a promising treatment option for psoriasis patients, especially those with refractory subtypes. It is a targeted therapy that delivers high-intensity UVB light to affected skin areas, promoting faster healing and reducing inflammation. The excimer laser has proven effective with minimal side effects, but careful patient selection is crucial to ensure optimal outcomes. It is most effective when used on localized areas of psoriasis, rather than widespread or generalized diseases. Patients with fair skin, psoriasis limited to a few areas, and those not responsive to other treatments are good candidates for this therapy. Despite the availability of highly efficacious biologic agents, the excimer laser can be used as an adjunct therapy with topical or systemic agents to improve the overall efficacy of treatment and reduce the risk of side effects associated with systemic agents.
